# Clinical and Immune Features of Hospitalized Pediatric Patients With Coronavirus Disease 2019 (COVID-19) in Wuhan, China

**DOI:** 10.1001/jamanetworkopen.2020.10895

**Published:** 2020-06-03

**Authors:** Huan Wu, Hongmin Zhu, Chunhui Yuan, Cong Yao, Wei Luo, Xin Shen, Jun Wang, Jianbo Shao, Yun Xiang

**Affiliations:** 1 Department of Laboratory Medicine, Wuhan Children’s Hospital, Tongji Medical College, Huazhong University of Science and Technology, Wuhan, China; 2 Department of Neurology, Wuhan Children’s Hospital, Tongji Medical College, Huazhong University of Science and Technology, Wuhan, China; 3 Health Care Department, Wuhan Children’s Hospital, Tongji Medical College, Huazhong University of Science and Technology, Wuhan, China; 4 Department of Clinical Laboratory, Tianjin Medical University General Hospital, Tianjin, China; 5 Department of Radiology, Wuhan Children’s Hospital, Tongji Medical College, Huazhong University of Science and Technology, Wuhan, China

## Abstract

**Importance:**

The epidemiologic and clinical characteristics of pediatric patients with coronavirus disease 2019 (COVID-19) have been reported, but information on immune features associated with disease severity is scarce.

**Objective:**

To delineate and compare the immunologic features of mild and moderate COVID-19 in pediatric patients.

**Design, Setting, and Participants:**

This single-center case series included 157 pediatric patients admitted to Wuhan Children’s Hospital with laboratory-confirmed severe acute respiratory syndrome coronavirus 2 (SARS-CoV-2). Data were collected from January 25 to April 18, 2020.

**Exposures:**

Documented SARS-CoV-2 infection.

**Main Outcomes and Measures:**

Clinical and immunologic characteristics were collected and analyzed. Outcomes were observed until April 18, 2020.

**Results:**

Of the 157 pediatric patients with COVID-19, 60 (38.2%) had mild clinical type with pneumonia, 88 (56.1%) had moderate cases, 6 (3.8%) had severe cases, and 3 (1.9%) were critically ill. The 148 children with mild or moderate disease had a median (interquartile range [IQR]) age of 84 (18-123) months, and 88 (59.5%) were girls. The most common laboratory abnormalities were increased levels of alanine aminotransferase (ALT) (median [IQR], 16.0 [12.0-26.0] U/L), aspartate aminotransferase (AST) (median [IQR], 30.0 [23.0-41.8] U/L), creatine kinase MB (CK-MB) activity (median [IQR], 24.0 [18.0-34.0] U/L), and lactate dehydrogenase (LDH) (median [IQR], 243.0 [203.0-297.0] U/L), which are associated with liver and myocardial injury. Compared with mild cases, levels of inflammatory cytokines including interleukin 6, tumor necrosis factor α, and interferon γ were unchanged, whereas the level of immune suppressive interleukin 10 was markedly increased in moderate cases compared with mild cases (median [IQR], 3.96 [3.34-5.29] pg/mL vs 3.58 [3.10-4.36] pg/mL; *P* = .048). There was no statistically significant difference in absolute number of lymphocytes (including T cells and B cells) between mild and moderate cases, but moderate cases were associated with a decrease in neutrophil levels compared with mild cases (median [IQR], 2310/μL [1680/μL-3510/μL] vs 3120/μL [2040/μL-4170/μL]; *P* = .01). Immunoglobin G and the neutrophil to lymphocyte ratio were negatively associated with biochemical indices related to liver and myocardial injury (immunoglobulin G, ALT: *r*, −0.3579; AST: *r*, −0.5280; CK-MB activity: *r*, −0.4786; LDH: *r*, −0.4984; and neutrophil to lymphocyte ratio, ALT: *r*, −0.1893; AST: *r*, −0.3912; CK-MB activity: *r*, −0.3428; LDH: *r*, −0.3234), while counts of lymphocytes, CD4^+^ T cells, and interleukin 10 showed positive associations (lymphocytes, ALT: *r*, 0.2055; AST: *r*, 0.3615; CK-MB activity: *r*, 0.338; LDH: *r*, 0.3309; CD4^+^ T cells, AST: *r*, 0.4701; CK-MB activity: *r*, 0.4151; LDH: *r*, 0.4418; interleukin 10, ALT: *r*, 0.2595; AST: *r*, 0.3386; CK-MB activity: *r*, 0.3948; LDH: *r*, 0.3794).

**Conclusions and Relevance:**

In this case series, systemic inflammation rarely occurred in pediatric patients with COVID-19, in contrast with the lymphopenia and aggravated inflammatory responses frequently observed in adults with COVID-19. Gaining a deeper understanding of the role of neutrophils, CD4^+^ T cells, and B cells in the pathogenesis of SARS-CoV-2 infection could be important for the clinical management of COVID-19.

## Key Points

**Question:**

What are the immunologic features of pediatric patients with pneumonia caused by coronavirus disease 2019 (COVID-19)?

**Findings:**

In this single-center case series involving 157 pediatric patients with COVID-19, systemic inflammation rarely occurred. Patients with moderate disease had higher interleukin 10 levels and lower neutrophil levels than patients with mild disease.

**Meaning:**

The results of this study suggest that dysregulation of immune response may be involved in the pathologic process of COVID-19; gaining a deeper understanding of the role of neutrophils, CD4^+^ T cells, and B cells in the pathogenesis of severe acute respiratory syndrome coronavirus 2 infection could be important for the clinical management of COVID-19.

## Teaser

This case series delineates and compares the immunologic features of mild and moderate coronavirus disease 2019 (COVID-19) in pediatric patients.

## Introduction

In December 2019, a highly infectious disease, ie, pneumonia caused by the coronavirus disease 2019 (COVID-19), occurred in Wuhan, Hubei Province, China, and the World Health Organization has declared its ongoing outbreak a pandemic.^1,2^ The pathogen of COVID-19, severe acute respiratory syndrome coronavirus 2 (SARS-CoV-2), had caused 3 090 445 confirmed cases and 217 769 deaths globally by May 1, 2020, according to a situation report from the World Health Organization.^3^

Previous studies in adult patients have confirmed that COVID-19 is more likely to affect older individuals with comorbidities,^4,5^ and the report by Zeng et al^6^ showed possible vertical transmission. A cohort study of 44 672 confirmed cases in China further reported that 1% to 2% were pediatric patients,^7^ and more than 90% of pediatric patients had mild or moderate disease.^8,9^ As the only designated hospital in Wuhan for treating children younger than 16 years with COVID-19, Wuhan Children’s Hospital has reported that the clinical course of pediatric patients has been milder compared with adults with the disease.^10^ However, the risk factors associated with disease severity of COVID-19 in pediatric patients remain unclear.

Analysis of clinical and immunologic characteristics has revealed that the lymphocyte counts are closely associated with severity of SARS-CoV-2 infection in adult patients, and 63% to 70% of patients with severe disease have lymphopenia and natural killer (NK) cell exhaustion, whereas the level of neutrophils, the main player of the so-called cytokine storm, was increased.^5,11,12^ Therefore, to facilitate efforts to prevent and control COVID-19 in children, we performed a comprehensive exploration of characteristics of 157 patients with laboratory-confirmed SARS-CoV-2 infection on admission to the hospital and compared the clinical and immune features of mild cases with moderate cases. These findings may help to extend our understanding of the risk factors associated with COVID-19 disease severity in pediatric patients.

## Methods

### Study Design and Participants

We performed a retrospective review of medical records of 157 pediatric patients admitted to Wuhan Children’s Hospital with laboratory-confirmed SARS-CoV-2 infection and a definite clinical outcome (ie, death or discharge) as of April 18, 2020. Wuhan Children’s Hospital is responsible for the treatment of pediatric patients with COVID-19, as assigned by the government. Diagnosis, clinical classifications, and complication definitions for COVID-19 were based on the *New Coronavirus Pneumonia Prevention and Control Program* (7th edition), published by the National Health Commission of China.^13^ All cases with COVID-19 tested positive for SARS-CoV-2 by use of real-time polymerase chain reaction assay either on throat or anal swab samples in Wuhan Children’s Hospital. The clinical outcomes (ie, discharges, mortality) were observed from January 25 to April 18, 2020.

This study was reviewed and approved by the medical ethical committee of Wuhan Children’s Hospital, Huazhong University of Science and Technology. All patients gave written consent (provided by at least a parent or guardian) to the passive use of their medical records for research purposes. The study followed the reporting guideline for case series.

### Collection of Clinical and Laboratory Data

We reviewed demographic, clinical, laboratory, treatment, and outcome data from patients’ electronic medical records. Clinical and laboratory data for each patient were collected before they received any treatment. All information was obtained and curated with a customized data collection form. Two of us (H.W. and H.Z.) independently reviewed the data collection forms to verify data accuracy.

Throat and anal swab samples were collected and tested for SARS-CoV-2 with the Chinese Center for Disease Control and Prevention recommended kit. All samples were processed at the Department of Laboratory Medicine of Wuhan Children’s Hospital. Total RNA was extracted within 2 hours using the nucleic acid isolation kit (DAAN Gene). The real-time reverse transcription–polymerase chain reaction assay was performed using a SARS-CoV-2 nucleic acid detection kit according to the manufacturer’s protocol (BGI Biotechnology). A cycle threshold value in FAM channel of 38 or less was defined as a positive test result, and a cycle threshold value of greater than 40 or no amplification curve was defined as a negative test result.

### Statistical Analysis

We present continuous variables as median (interquartile range [IQR]) or mean (SD) and categorical variables as number and percentage. Statistical differences for continuous variables were compared using unpaired *t* tests when the data were normally distributed; otherwise, the Mann-Whitney U test was used. Proportions for categorical variables were compared using the χ^2^ test or the Fisher exact test. All statistical analyses were performed using SPSS statistical software version 26.0 (IBM Corp). Spearman correlation analysis between the immune-associated biomarkers and biochemical indexes was conducted using Prism version 6.00 (GraphPad ). A 2-sided α < .05 was considered statistically significant.

## Results

### Demographic Characteristics and Baseline Clinical Features of Pediatric Patients With Mild and Moderate COVID-19

As of April 18, 2020, a total of 157 pediatric patients were confirmed to have SARS-Cov-2 infection by reverse transcription–polymerase chain reaction assay in Wuhan Children’s Hospital. According to the guidelines for diagnosis and management of COVID-19 issued by the National Health Commission of China, 60 (38.2%) had mild disease with pneumonia, 88 (56.1%) had moderate disease, 6 (3.8%) had severe disease, and 3 (1.9%) were critically ill, of whom 2 (66.7%) had coexisting conditions (ie, leukemia [for which the patient was receiving maintenance chemotherapy] and intussusception) and died. The others were discharged. Demographic data and baseline clinical features of mild and moderate cases are summarized in Table 1. The children with mild or moderate disease had a median (IQR) age of 84 (18-123) months, and 88 (59.5%) were girls. The median (IQR) age of children with moderate disease (66 [8-117] months) was significantly younger than that of children with mild cases (108 [46-136] months; *P* = .003). SARS-CoV-2 reverse transcription–polymerase chain reaction results became negative after a median (IQR) of 7 (4-11) days of treatment, except for the 2 patients who died, who had persistently positive results.

**Table 1.** Demographic and Clinical Characteristics of Pediatric Patients With Coronavirus Disease 2019 on Admission to the Hospital

Characteristic	No. (%)			*P* value^a^
	Total (N = 148)	Mild (n = 60)	Moderate (n = 88)	
Age, median (IQR), mo	84 (18-123)	108 (46-136)	66 (8-117)	.003
Sex				
Girls	88 (59.5)	33 (55.0)	55 (62.5)	.36
Boys	60 (40.5)	27 (45.0)	33 (62.5)	
Time from onset to hospital admission, median (IQR), d	5 (2-7)	5 (2-7)	5 (2.5-7)	.83
Signs and symptoms				
None	45 (30.4)	32 (53.3)	13 (14.8)	<.001
Fever	60 (40.5)	15 (25.0)	45 (51.1)	.002
Dry cough	66 (44.6)	18 (30.0)	48 (54.5)	.003
Vomiting or diarrhea	32 (21.6)	6 (10.0)	26 (29.5)	.005
Headache	5 (3.4)	1 (1.7)	4 (4.5)	.65
Other, eg, milk choking, spit up, somnolence	43 (29.1)	6 (10.0)	37 (42.0)	<.001
CT evidence of pneumonia				
Unilateral	54 (36.5)	0	54 (61.4)	NA
Bilateral	34 (23.0)	0	34 (38.6)	
Ground-glass opacity	51 (34.5)	0	51 (58.0)	
Treatment				
Antiviral therapy	147 (99.3)	59 (98.3)	88 (100)	NA
Antibiotic therapy	68 (45.9)	20 (33.3)	48 (54.5)	.01
Intensive unit care	0	0	0	NA
Invasive mechanical ventilation	0	0	0	NA
Clinical outcome				
Discharged	148 (100)	60 (100)	88 (100)	NA
Time from positive to negative for PCR assay, median (IQR), d	7 (4-11)	8 (4-12)	6 (4-10)	.27
Died	0	0	0	NA

Abbreviations: CT, computed tomography; IQR, interquartile range; NA, not applicable; PCR, polymerase chain reaction.

^a^ *P* values compare mild cases and moderate cases using χ^2^ test, Fisher exact test, or Mann-Whitney test.

### Difference of Organ Function and Infection-Associated Biomarkers Between Pediatric Patients With Mild and Moderate COVID-19

Compared with the reference range, 12 pediatric patients (7.6%) had increased alanine aminotransferase (ALT) levels on admission (median [IQR], 16.0 [12.0-26.0] U/L [to convert to microkatals per liter, multiply by 0.0167]), 25 (16.9%) had increased aspartate aminotransferase (AST) levels on admission (median [IQR], 30.0 [23.0-41.8] U/L [to convert to microkatals per liter, multiply by 0.0167]), 64 (40.8%) had increased serum creatine kinase MB (CK-MB) activity levels on admission (24.0 [18.0-34.0] U/L [to convert to microkatals per liter, multiply by 0.0167]), 32 (21.6%) had increased lactate dehydrogenase (LDH) levels on admission (median [IQR], 243.0 [203.0-297.0] U/L [to convert to microkatals per liter, multiply by 0.0167]), 48 (32.4%) had increased C-reactive protein levels on admission, and 70 (47.3%) had increased procalcitonin levels on admission. Table 2 presents findings of laboratory examinations related to coagulation, cardiac, liver, and renal damage according to mild or moderate disease. Some features differed significantly between mild and moderate cases of COVID-19, including increased levels of dimerized plasmin fragment D (median [IQR], 0.16 [0.13-0.26] μg/mL vs 0.24 [0.15-0.36] μg/mL [to convert to nanomoles per liter, multiply by 5.476]; *P* = .02), ALT (median [IQR], 13.0 [11.0-18.8] U/L vs 18.0 [12.3-33.8] U/L; *P* = .001), AST (median [IQR], 25.0 [20.3-34.5] U/L vs 33.0 [24.0-46.8] U/L; *P* < .001), γ-glutamyltransferase (median [IQR], 10.0 [8.0-13.0] U/L vs 12.0 [10.0-19.0] U/L [to convert to microkatals per liter, multiply by 0.0167]; *P* = .005), and LDH (median [IQR], 222.0 [190.3-269.8] U/L vs 254.0 [218.5-309.0] U/L; *P* = .004) and decreased levels of total bilirubin (median [IQR], 0.51 [0.36-0.67] mg/dL vs 0.43 [0.29-0.58] mg/dL [to convert to micromoles per liter, multiply by 17.104]; *P* = .04) and creatinine (median [IQR], 0.43 [0.32-0.51] mg/dL vs 0.35 [0.26-0.48] mg/dL [to convert to micromoles per liter, multiply by 88.4]; *P* = .02).

**Table 2.** Difference of Laboratory Findings of Pediatric Patients With Coronavirus Disease 2019 on Admission to Hospital

Biomarker	Median (IQR)			*P* value^a^
	Total (N = 148)	Mild (n = 60)	Moderate (n = 88)	
Coagulation function				
Prothrombin time, s	10.9 (10.5-11.3)	10.9 (10.6-11.4)	10.8 (10.5-11.1)	.24
Fibrinogen, g/L	207 (177-252)	199 (176-231)	214 (178-267)	.26
Activated partial thromboplastin time, s	30.8 (28.7-33.8)	30.3 (27.8-33.5)	30.9 (28.8-34.2)	.28
Thrombin time, s	18.4 (17.7-19.4)	18.6 (18.0-19.2)	18.4 (17.5-19.6)	.84
D-dimer, μg/mL	0.20 (0.14-0.35)	0.16 (0.13-0.26)	0.24 (0.15-0.36)	.02
Liver function				
Total bilirubin, mg/dL	0.44 (0.32-0.61)	0.51 (0.36-0.67)	0.43 (0.29-0.58)	.04
Direct bilirubin, mg/dL	0.14 (0.10-0.19)	0.15 (0.10-0.20)	0.13 (0.09-0.19)	.12
Albumin, g/dL	4.54 (4.32-4.77)	4.56 (43.4-48.2)	4.54 (4.31-4.75)	.27
Globulin, mean (SD), g/dL	2.30 (0.48)	2.34 (0.37)	2.27 (0.54)	.48
Alanine aminotransferase, U/L	16.0 (12.0-26.0)	13.0 (11.0-18.8)	18.0 (12.3-33.8)	.001
γ-glutamyltransferase, U/L	11.0 (9.0-16.0)	10.0 (8.0-13.0)	12.0 (10.0-19.0)	.005
Aspartate aminotransferase				
Level, U/L	30.0 (23.0-41.8)	25.0 (20.3-34.5)	33.0 (24.0-46.8)	<.001
Increased, No. (%)	25 (16.9)	4 (6.7)	21 (23.9)	.007
Renal function				
Creatinine, mg/dL	0.38 (0.29-0.50)	0.43 (0.32-0.51)	0.35 (0.26-0.48)	.02
Blood urea nitrogen, mean (SD), mg/dL	11.2 (3.64)	11.7 (3.5)	10.9 (3.7)	.15
Uric acid, mg/dL	0.41 (0.34-0.53)	0.44 (0.35-0.53)	0.41 (0.33-0.54)	.81
Cystatin C, mg/L	0.99 (0.93-1.12)	0.99 (0.91-1.07)	1.01 (0.93-1.13)	.22
Retinol binding protein, μg/mL	22.15 ± 4.71	21.95 ± 4.50	22.21 ± 4.84	.27
Myocardial zymogram				
Creatine kinase, U/L	102.0 (75.5-137.0)	100.5 (69.0-125.5)	105.0 (78.0-147.5)	.35
Creatine kinase MB activity, U/L	24.0 (18.0-34.0)	20.0 (17.0-29.5)	25.0 (19.0-34.0)	.06
Lactate dehydrogenase				
Level, U/L	243.0 (203.0-297.0)	222.0 (190.3-269.8)	254.0 (218.5-309.0)	.004
Increased, No. (%)	32 (21.6)	10 (16.7)	22 (25.0)	.23
Infection				
C-reactive protein				
Normal, <0.075 mg/dL	100 (67.6)	46 (76.7)	54 (61.4)	.05
Increased	48 (32.4)	14 (23.3)	34 (38.6)	NA
Procalcitonin				
Level, ng/mL	0.05 (0.04-0.08)	0.05 (0.04-0.07)	0.05 (0.04-0.08)	.55
Increased, No. (%)	70 (47.3)	24 (40.0)	46 (52.3)	.14
Ferritin, ng/mL	57.12 (40.7-86.03)	56.45 (36.98-76.03)	58.05 (42.88-103.40)	.21

Abbreviations: D-dimer, fibrin degradation product D; IQR, interquartile range; NA, not applicable.

SI conversion factors: To convert alanine aminotransferase, aspartate aminotransferase, creatine kinase, creatine kinase MB activity, γ-glutamyltransferase, lactate dehydrogenase to microkatals per liter, multiply by 0.0167; albumin and globulin to grams per liter, multiply by 10; C-reactive protein to milligrams per liter, multiply by 10; creatinine to micromoles per liter, multiply by 88.4; D-dimer to nanomoles per liter, multiply by 5.476; direct and total bilirubin to micromoles per liter, multiply by 17.104; ferritin to micrograms per liter, multiply by 1.0; fibrinogen to grams per liter, multiply by 0.01; urea nitrogen to millimoles per liter, multiply by 0.357; and uric acid to millimoles per liter, multiply by 0.0595.

^a^ *P* values comparing mild cases and moderate cases are from Mann-Whitney test or unpaired *t* test.

### Immunologic Features of Mild and Moderate Pediatric Patients With COVID-19

Compared with reference range, 16 children (10.2%) had mild leucopenia on admission and 13 (8.3%) had increased neutrophil levels. Counts of lymphocytes were decreased in only 7 cases (4.5%), which may be owing to decreased NK cells in 35 cases (22.3%), given that T cells and B cells were barely changed. Only 3 (1.9%) had decreased levels of CD4^+^ T cells, and 14 children with moderate cases (15.9%) had increased levels of CD4^+^ T cells. Levels of serum cytokines on admission, including interleukin (IL) 2, IL-4, IL-6, tumor necrosis factor α (TFN-α), and interferon γ (IFN-γ) were rarely increased, except that 1 critically ill patient with underlying intussusception had an IL-6 level of 3868.86 pg/mL. Immunosuppressive cytokine IL-10 increased in 22 cases (14.0%). These results suggest that systemic inflammation rarely occurred in pediatric patients.

**Figure 1.**
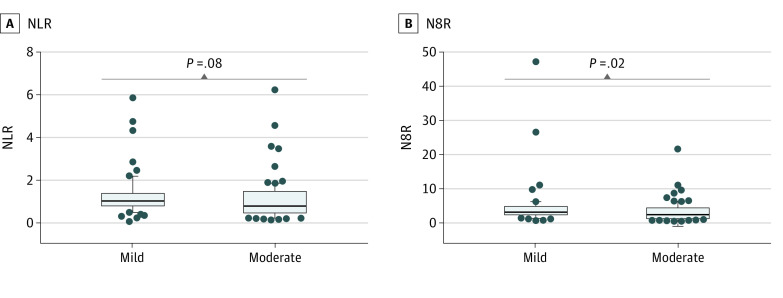
Neutrophil to Lymphocyte Ratio (NLR) and Neutrophil to CD8^+^ T Cell Ratio (N8R) in Pediatric Patients With Coronavirus Disease 2019 Comparisons of NLR with N8R between 60 mild cases and 88 moderate cases. Boxes represent medians and interquartile ranges, with whiskers indicating 10% to 90% range. Dots represent patients who fell outside the 10% to 90% range. Data were analyzed using Mann-Whitney test.

Table 3 presents blood cell counts, inflammatory cytokines, immunoglobulins (Igs), complement proteins, and lymphocyte subsets according to mild or moderate disease. Compared with mild cases, levels of IL-10 (median [IQR], 3.58 [3.10-4.36] pg/mL vs 3.96 [3.34-5.29] pg/mL; *P* = .048), C4 complement (median [IQR], 18 [13-23] mg/dL vs 22 [16-30] mg/dL; *P* = .001), and NK cells (median [IQR], 316 [160-477] n/μL vs 390 [270-543] n/μL; *P* = .048) were higher in moderate cases, while levels of IgG (median [IQR], 985 [842-1183] mg/dL vs 889 [550-1118] mg/dL [to convert to grams per liter, multiply by 0.01]; *P* = .02), counts of neutrophils (median [IQR], 3120/μL [2040/μL-4170/μL] vs 2310/μL [1680/μL-3510/μL] [to convert to ×10^9^/L, multiply by 0.001]; *P* = .01) and basophils (median [IQR], 20/μL [10/μL-30/μL] vs 10/μL [10/μL-20/μL] [to convert to ×10^9^/L, multiply by 0.001]; *P* = .01), and the neutrophil to CD8^+^ T cell ratio (N8R) (median [IQR], 3.14 [2.38-4.88] vs 2.45 [1.44-4.35]; *P* = .02) were significantly deceased (Table 3 and Figure 1). Counts of T cells and B cells and the neutrophil to lymphocyte ratio (NLR) showed no difference (Table 3 and Figure 1).

**Table 3.** Difference of Immune Features Between Pediatric Patients With Mild and Moderate Coronavirus Disease 2019 on Admission to Hospital

Biomarker	Median (IQR)			*P* value^a^
	Total (N = 148)	Mild (n = 60)	Moderate (n = 88)	
IL-2, pg/mL	1.46 (1.23-1.72)	1.42 (1.21-1.84)	1.47 (1.32-1.71)	.40
IL-4, pg/mL	2.70 (2.07-3.36)	2.72 (2.07-3.51)	2.58 (2.06-3.25)	.60
IL-6, pg/mL	3.85 (2.97-6.12)	3.87 (2.73-5.73)	3.83 (3.19-6.60)	.51
IL-10, pg/mL	3.84 (3.21-4.91)	3.58 (3.10-4.36)	3.96 (3.34-5.29)	.048
TNF-α, pg/mL	1.62 (1.26-2.23)	1.62 (1.16-2.32)	1.62 (1.28-2.20)	.85
IFN-γ, pg/mL	3.00 (2.27-4.62)	2.92 (2.12-4.71)	3.01 (2.35-4.54)	.92
IgG, mg/dL	945 (682-1153)	985 (842-1183)	889 (550-1118)	.02
IgA, mg/dL	121 (37-173)	123 (72-168)	110 (25-212)	.45
IgM, mg/dL	90 (60-117)	90 (63-120)	88 (51-116)	.63
IgE, mg/dL	0.0086 (0.0029-0.0277)	0.0089 (0.0053-0.0292)	0.0086 (0.0024-0.0244)	.61
C3 complement, mg/dL	91 (82-108)	92 (82-101)	90 (82-109)	.70
C4 complement, mg/dL	21 (15-27)	18 (13-23)	22 (16-30)	.001
White blood cells, /μL	6770 (5510-8230)	6850 (5750-8080)	6670 (5390-8520)	.40
Neutrophils, /μL	2600 (1810-3780)	3120 (2040-4170)	2310 (1680-3510)	.01
Lymphocytes, /μL	2990 (2360-4170)	2920 (2530-3430)	3040 (2180-4860)	.56
Monocytes, /μL	440 (350580)	430 (340-560)	440 (360-610)	.66
Eosinophils, /μL	110 (60-200)	110 (60-220)	110 (60-170)	.66
Basophils, /μL	20 (10-20)	20 (10-30)	10 (10-20)	.01
CD3^+^ T cells, n/μL	2419 (1869-3059)	2249 (1777-2711)	2525 (1887-3614)	.10
CD3^+^CD4^+^ T cells, n/μL	1144 (848-1727)	1117 (834-1492)	1237 (892-1900)	.14
CD3^+^CD8^+^ T cells, n/μL	973 (707-1249)	963 (674-1181)	1014 (710-1281)	.30
CD3^+^CD4^+^CD8^+^ T cells, n/μL	11 (6-22)	11 (6-22)	12 (5-22)	.91
Natural killer cells, n/μL	374 (212-509)	316 (160-477)	390 (270-543)	.048
CD19^+^ B cells, n/μL	635 (401-1021)	564 (420-710)	764 (389-1201)	.13

Abbreviations: IFN-γ, interferon γ; Ig, immunoglobulin; IL, interleukin; IQR, interquartile range; TNF-α, tumor necrosis factor α.

SI conversion factors: To convert basophils, eosinophils, lymphocytes, monocytes, neutrophils, and white blood cells to ×10^9^ per liter, multiply by 0.001; C3 complement, C4 complement, IgA, IgG, and IgM to grams per liter, multiply by 0.01; and IgE to mg/L, multiply by 10.

^a^ *P* values comparing mild cases and moderate cases are from Mann-Whitney test.

### Correlation Analysis Between Immunologic Features and Organ Function Related Biomarkers

**Figure 2.**
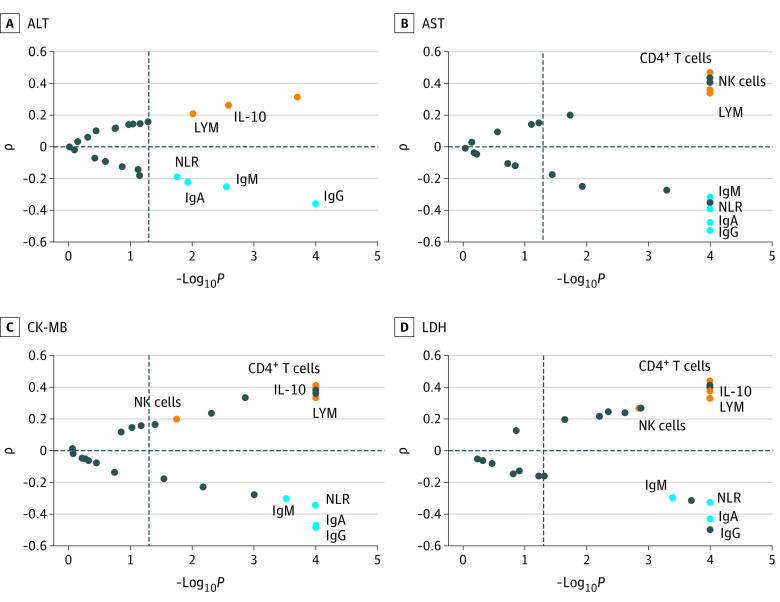
Spearman Rank Correlation Coefficient (ρ) Analysis Between Immune Features and Biochemical Indexes Orange dots indicate a positive association, light blue dots indicate a negative association, and dark blue dots indicate other immune indices. ALT indicates alanine aminotransferase; AST, aspartate aminotransferase; CK-MB, creatine kinase MB; Ig, immunoglobulin; IL-10, interleukin 10; LDH, lactate dehydrogenase; LYM, lymphocytes; NK, natural killer; NLR, neutrophil to lymphocyte ratio.

The most common abnormal results from laboratory tests observed in this study were associated with liver and myocardial injury, including ALT, AST, CK-MB activity, and LDH. To examine the role of immune responses in organ injury among pediatric patients with COVID-19, Spearman rank correlation coefficient analysis was performed between immunologic features and these 4 biochemical indexes (Figure 2). We found negative correlations between ALT, AST, CK-MB activity, and LDH with IgG (ALT: *r*, −0.3579; AST: *r*, −0.5280; CK-MB activity: *r*, −0.4786; LDH: *r*, −0.4984), IgM (ALT: *r*, −0.2480; AST: *r*, −0.3164; CK-MB activity: *r*, −0.3012; LDH: *r*, −0.2929), IgA (ALT: *r*, −0.2200; AST: *r*, −0.4753; CK-MB activity: *r*, −0.4685; LDH: *r*, −0.4223), and the NLR (ALT: *r*, −0.1893; AST: *r*, −0.3912; CK-MB activity: *r*, −0.3428; LDH: *r*, −0.3234), while we found positive correlations with counts of NK cells (ALT: *r*, 0.3113; AST: *r*, 0.3622; CK-MB activity: *r*, 0.2009; LDH: *r*, 0.2684), lymphocytes (ALT: *r*, 0.2055; AST: *r*, 0.3615; CK-MB activity: *r*, 0.338; LDH: *r*, 0.3309), and CD4^+^ T cells (AST: *r*, 0.4701; CK-MB activity: *r*, 0.4151; LDH: *r*, 0.4418) as well as IL-10 levels (ALT: *r*, 0.2595; AST: *r*, 0.3386; CK-MB activity: *r*, 0.3948; LDH: *r*, 0.3794).

## Discussion

The clinical features of pediatric patients with COVID-19 have been reported to be much milder than those of adults.^8,9,10^ However, there is insufficient knowledge regarding the immunologic features related to the clinical outcomes of COVID-19 in pediatric patients. The findings of our study show that systemic inflammation rarely occurred in pediatric patients. Among the immune cell subgroups, lymphocytes (including T cells and B cells) were increased and neutrophils were decreased with COVID-19 progression. Specifically, we observed that NLR was negatively associated with ALT, AST, CK-MB activity, and LDH levels, the most common abnormal laboratory test results among pediatric patients with COVID-19. These data were in contrast with the results obtained from adult patients, which showed significantly increased NLR among adult patients, helpful for the early screening of critical cases.^14,15,16^ A possible explanation may be that aging is associated with increased neutrophil accumulation during viral infection, and excessive neutrophil responses induce tissue injury and worsen disease. Previous studies have reported that recruitment of neutrophils into influenza-infected trachea is essential for CD8^+^ T cell–mediated immune protection in mice, while aging increases mortality from influenza because of chemokines secreted by senescent alveolar epithelial cells, leading to excessive neutrophil recruitment.^17,18^ Thus, we suggest that age-related neutrophil recruitment may be why COVID-19 is milder in pediatric patients than in adult patients.

Although a decrease of CD4^+^ T cells was common in adult patients with severe and moderate COVID-19,^16^ this was rarely seen in pediatric patients (3 [1.9%]), and even increased in 14 moderate cases (15.9%). These results may be because CD4^+^ T cell–derived IL-10 was most important for calming inflammation and because of the maturation of memory CD8^+^ T cells during the resolution phase of viral infection.^19^ Indeed, counts of CD4^+^ T cells and IL-10 were positively associated with biomarkers associated with liver and myocardial injury in pediatric patients. Taking all these findings into consideration, neutrophils may play an important role in the initial phase, and CD4^+^ T cells may contribute to the resolution phase of SARS-CoV-2 infection. This may be why N8R significantly decreased in moderate cases but was not associated with liver and myocardial enzymes. Nevertheless, the role of neutrophils and CD4^+^ T cells during the development of COVID-19 warrants further investigation.

Research in adult patients has highlighted the importance of T lymphocytes, CD4^+^ T cells in particular, in controlling and fine-tuning the pathogenesis and outcomes of SARS-CoV and Middle East respiratory syndrome CoV infection.^15^ However, the function of B cells and antibodies is often ignored despite the fact that B cells also decreased among patients with COVID-19.^20^ In this study, the serum concentration of IgG was significantly decreased in moderate cases compared with mild cases. Notably, Igs, including IgG, IgA, and IgM, were negatively associated with biomarkers associated with liver and myocardial injury in pediatric patients. It is reported that antibodies to the neuraminidase are the major mediators of protection against influenza virus infection and display broad binding activity, spanning the entire history of influenza A virus circulation in humans, including the original pandemic strains of both H1N1 and H3N2 subtypes.^21^ Furthermore, we observed that titers and duration time of IgG to SARS-CoV-2 in pediatric patients showed no significant difference compared with adult patients (H. Xiao, PhD, unpublished data, 2020). These data suggest that B cells also play an important role in controlling SARS-CoV-2 infection. Further research is required to determine the influence of B cells in the context of COVID-19.

## Limitations

There were several limitations in our study that might create bias. First, it was a retrospective and single-center study of patients admitted to the hospital; standardized data for a larger, multicenter cohort would be better to assess the temporal change of immune response after SARS-COV-2 infection. Second, patients with COVID-19 who have *Mycoplasma* coinfection or superinfection might affect the results of immune response, which is a common cause of pneumonia in children and occurred in 40 cases in this study.

## Conclusions

To our knowledge, this is the first study to describe the changes of lymphocyte subsets and cytokine profiles in pediatric patients with COVID-19. Our study showed that systemic inflammation rarely occurred in pediatric patients, different from the lymphopenia and aggravated inflammatory responses frequently observed in adults with COVID-19. Gaining a deeper understanding of the role of neutrophil, CD4^+^ T cells, and B cells in the pathogenesis of SARS-CoV-2 infection could be important for the clinical management of COVID-19.
